# Reactivity of Low-Grade Chromite Concentrates towards Chlorinating Atmospheres

**DOI:** 10.3390/ma13204470

**Published:** 2020-10-09

**Authors:** Ndue Kanari, Eric Allain, Lev Filippov, Seit Shallari, Frédéric Diot, Fabrice Patisson

**Affiliations:** 1 Université de Lorraine, CNRS, GeoRessources, F-54000 Nancy, France; ericgallain@gmail.com (E.A.); lev.filippov@univ-lorraine.fr (L.F.); frederic.diot@univ-lorraine.fr (F.D.); 2 Faculty of Agriculture and Environment, Agricultural University of Tirana, 1029 Tirana, Albania; seitshallari@gmail.com; 3 Université de Lorraine, CNRS, Labex DAMAS, IJL, F-54000 Nancy, France; fabrice.patisson@univ-lorraine.fr

**Keywords:** chromite, chlorine, thermogravimetric analysis, isothermal treatment, apparent activation energy

## Abstract

The most economically important iron-chromium bearing minerals is chromite. In natural deposits, iron(II) is frequently substituted by magnesium(II) while chromium(III) is replaced by aluminum(III) and/or iron(III) forming a complex chromium bearing material. The majority of mined chromite is intended for the production of ferrochrome which requires a chromite concentrate with high chromium-to-iron ratio. Found mostly in the spinel chromite structure, iron cannot be removed by physical mineral processing methods. In this frame, the present work deals with the reaction of chlorine and chlorine+oxygen with selected samples of chromite concentrates for assessing the reactivity of their components towards chlorinating atmosphere, allowing the preferential removal of iron, hence meeting the chromite metallurgical grade requirements. Isothermal thermogravimetric analysis was used as a reliable approach for the kinetic reactivity investigation. Results indicated a wide difference in the thermal behavior of chromite constituents in a chlorinating atmosphere when considering their respective values of apparent activation energy oscillating from about 60 to 300 kJ/mol as a function of the sample reacted fraction. During the chromite treatment by chlorine in presence of oxygen, chromium was recovered as liquid chromyl chloride by condensation of the reaction gas phase.

## 1. Introduction

**Figure 1:**
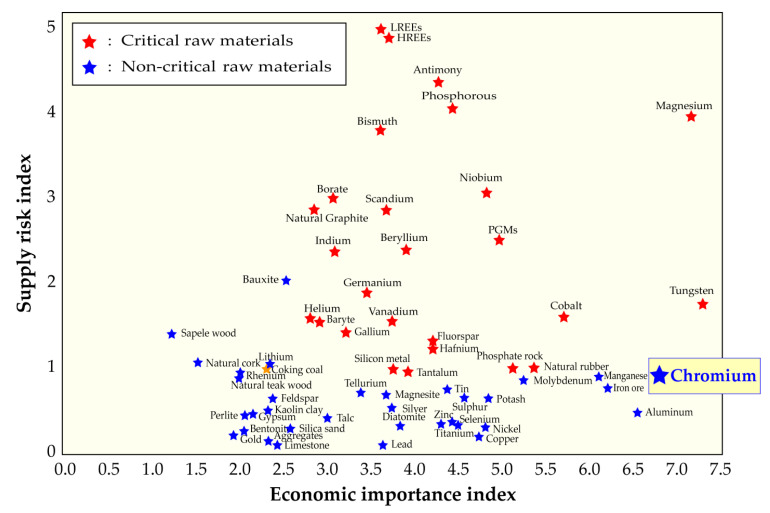
The 2017 criticality assessment of raw materials for the European Union.

Chromium is part of the extended group of refractory metals offering beneficial properties for various end-uses and manufacturing applications. As displayed in Figure 1, part of these metals (Nb, W, V, Hf, Ta) belongs to the critical materials, according to the European Union criticality assessment [1]. Although chromium is in the cut-off level to be critical, it ranks third (after Mg and W) from the standpoint of economic importance.

Most refractory metals are found and extracted from their oxide bearing materials, likewise, the only economic source of chromium is chromite (FeCr_2_O_4_) ore. Nevertheless, in natural deposits, Mg(II) may substitute Fe(II), while Al(III) and Fe(III) often substitute Cr(III) resulting to a complex chromium bearing mineral with the fairly general formula (Fe,Mg)(Cr,Al,Fe)_2_O_4_, encompassing the main end-members such as FeO·Fe_2_O_3_ (magnetite), FeO·Cr_2_O_3_ (iron chromite), MgO·Cr_2_O_3_ (magnesiochromite), MgO·Al_2_O_3_ (true/regularly spinel). Being multiple and complete solid solution of the spinel group, the composition of chromite is no fixed, but varies largely and depends on the geographic and geochemical features of its deposits.

The major part of mined chromite goes to the ferrochrome (FeCr) manufacture [2,3,4,5] and in turn, the FeCr is intended to stainless steels and chromium bearing alloys production. It seems that chromium has no substitute for these industrial end-uses. According to available data [6], the average annual growth of the world stainless steels production is about 5.84% reaching 50.7 million metric tons in 2018. Selected reports [7,8,9,10,11,12,13] from numerous recent research works reported to the scientific journals *Materials* and *Metals* are focused on the chromium bearing steels and alloys showing the importance of these leading areas for chromium utilization driving the needs for the ferrochrome and chromite production. Chemical industry, foundry sands and refractory segments are other end-uses of chromite, but their weight relative to the total chromite demand is minor.

It must be emphasized that the production of ferrochrome not only requires a chromite ore and/or concentrate with a high Cr_2_O_3_ content (46–48% Cr_2_O_3_), but also with a chromium-iron ratio above 2 (typically around 2.8). The physical processing of the mineral can be successful for the removal of the chromite ore gangue leading to a concentrate with a satisfactory Cr_2_O_3_ content meeting the metallurgical requirement. However, as the major part of iron is found in the lattice structure of chromite, only a chemical broken down of the chromite structure seems appropriate for the removal of iron from chromite. According to Nafziger [14], chemical techniques, such as hydrometallurgical methods, chlorination, roasting and leaching, as well as smelting, are required for increasing the chromium-to-iron ratio of lean chromite ores and concentrates. Recently, a carbo-thermic reduction followed by hydrochloric acid leaching was tried as an efficient method [15] for the extraction of iron from a poor chromium concentrate. Specific research works [16,17,18,19,20,21] regarding the use of various chlorination agents for chlorination of chromite ores and concentrate and their constituents were summarized earlier [2,3].

Our various research reports published previously [2,3,22,23,24] were focused on the carbochlorination, chlorination and oxychlorination of rich chromite concentrates having a chromium-to-iron ratio of around 3.2 suitable for ferrochrome manufacturing. However, no recent studies were disclosed in the literature regarding to the use of Cl_2_+O_2_ for processing lean chromite materials, i.e., having typically a chromium to iron ratio lesser than 2.8. In this regard, the present paper essentially describes the behavior of a poor chromite concentrate with a chromium-to-iron ratio near to 1.5, under Cl_2_ and Cl_2_+air atmospheres. The reactivity and behavior of chromite constituents were examined using thermogravimetric analysis (TGA) approach under isothermal conditions. For a better understanding of the processes, the experimental results are compared with those obtained for the treatment of a rich chromite concentrate (of metallurgical grade).

## 2. Materials and Methods

The first chromite concentrate sample (low grade) used for this investigation was provided by a European Union manufacturer. A second sample (high grade) provided by an Albanian chromite plant (Bulqiza, Albania) was also used, mostly for comparison purpose. The physico-chemical characterization of the chromite samples and of the reaction products was performed by diverse analytical methods such as chemical analysis (by inductively coupled plasma atomic emission spectroscopy “ICP-AES”), scanning electron microscopy-energy dispersive spectroscopy (SEM-EDS, HITACHI S-4800, Hitachi Ltd., Tokyo, Japan) and X-ray diffraction (XRD, Bruker D8 Advance device, Bruker, Karlsruhe, Germany). Their description was given in previous research works [2,25,26]; only the results will be reported hereafter.

**Figure 2:**
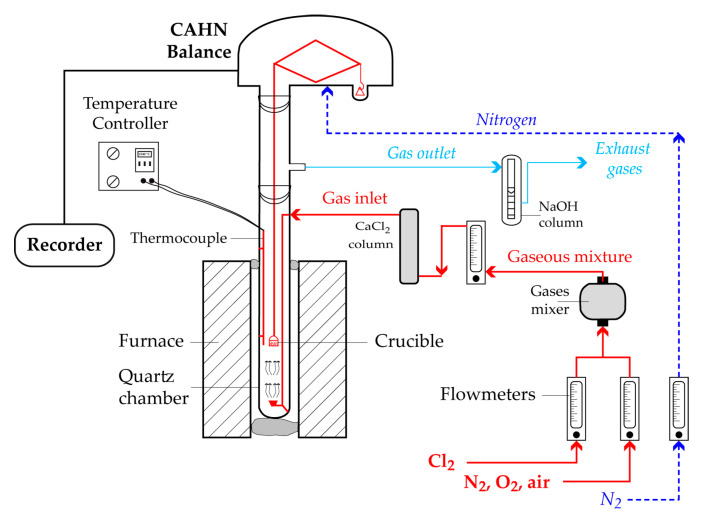
Setup of the TG analysis experiment.

Experimental tests of the reaction of chromite concentrates with chlorine were carried out in a vertical microbalance (model CAHN 1000, Cahn Co., Cerritos, CA, USA) operating at a sensitivity of 10 μg and designed to work under corrosive atmosphere. The equipment configuration with the accessory parts is shown schematically in Figure 2. Several milligrams (more often 40 to 50 mg) of sample were spread out in a silica crucible and the whole specimen was heated up to the desired temperature under nitrogen atmosphere. Subsequently, the nitrogen was replaced by Cl_2_ and/or Cl_2_+air (O_2_) and the evolution of the mass loss over time was recorded. The accessory parts of the setup illustrated in Figure 2 are the units for measuring and purifying the inlet gases as well as those for neutralizing the outlet gases.

## 3. Results

### 3.1. Physico-Chemical Characterization of Chromite Concentrate Samples

**Figure 3:**
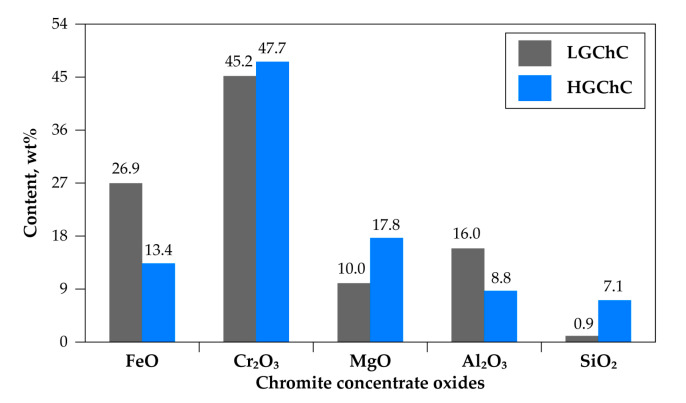
Chemical composition of the chromite concentrate samples.

**Figure 4:**
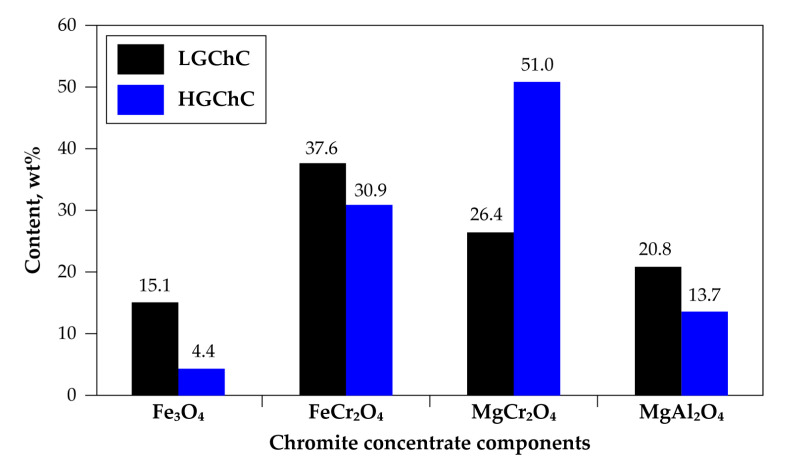
Mineralogical composition of the chromite concentrate samples.

The chemical composition in the five main constituents of the first chromite sample is given in Figure 3. As shown, this concentrate is characterized by a high iron content (26.9% wt expressed as FeO) and its chromium-to-iron ratio of 1.48 makes it unsuitable for the FeCr manufacturing. This sample is denoted as low-grade chromite concentrate (LGChC). The XRD patterns of the sample matched well with the (Fe,Mg)(Cr,Fe,Al)_2_O_4_ phase. Note that the simple constituents of chromite (Fe_3_O_4_, FeCr_2_O_4_, MgCr_2_O_4_ and MgAl_2_O_4_) are isomorphs having analogous XRD profile making their individual identification difficult. Based on the chemical analysis and supposing a perfect stoichiometric composition without cation/anion deficiency and/or defect, the general formula of the chromite body of LGChC can be approximately represented as (Fe_0.50_,Mg_0.50_)(Cr_1.20_,Al_0.60_,Fe_0.20_)O_4_ and the chromite body can also be expressed as 15.1% Fe_3_O_4_, 37.6% FeCr_2_O_4_, 26.4% MgCr_2_O_4_ and 20.8% MgAl_2_O_4_ (Figure 4).

The second sample had a chromium-to-iron ratio close to 3.2 and is labelled as high-grade chromite concentrate (HGChC). Although a rich concentrate with 47.7% Cr_2_O_3_ (Figure 3), it contained high amount of gangue (close to 7% SiO_2_ belonging also to olivine and serpentine minerals). The chromite body was separated from the gangue by successive physical separations (using dense liquor) and was defined as (Fe_0.30_, Mg_0.70_)(Cr_1.56_,Al_0.37_,Fe_0.07_)O_4_ with the average composition of end-members 4.4% Fe_3_O_4_, 30.9% FeCr_2_O_4_, 51.0% MgCr_2_O_4_ and 13.7% MgAl_2_O_4_ (Figure 4). The mean particle size of both chromite samples used for this study is less than 100 μm. As the reactions of HGChC with Cl_2_+CO, Cl_2_ and Cl_2_+O_2_ gaseous mixtures were studied thoroughly in previous research work [2,3,22,23,24], its behaviour in the chlorinating atmosphere is used as a reference to explain the phenomena observed during the treatment of the low-grade chromite concentrate.

### 3.2. Behavior of Chromite under Chlorine Atmosphere

Envisaged reactions of the complex chromite constituents (Fe_3_O_4_, FeCr_2_O_4_, MgCr_2_O_4_ and MgAl_2_O_4_) and those of simple oxides (FeO, Fe_2_O_3_, Cr_2_O_3_, MgO and Al_2_O_3_) with chlorine may be represented by Equation (1) through (9). The value of standard free energy changes (Δ_r_G°) at 900 °C is computed from HSC thermochemical database [27] and is indicated beside each reaction. According to these values, the reactions of chromite constituents with Cl_2(g)_ (Equations (1)–(4)) proceed with Δ_r_G° > 0 indicating a nonspontaneous process in the forward direction; these reactions will absorb energy from its surroundings in order to take place. Among the reactions of simple metals oxides of the chromite with chlorine involving their respective chlorides (Equations (5) to (9)), only the reaction of FeO seems to be a spontaneous reaction from a thermodynamic point of view; Cr_2_O_3_ and Al_2_O_3_ are the most stable oxides in chlorine atmosphere.

Δ_r_G° (900 °C), kJ/mol Cl_2_
2/9 Fe_3_O_4(s)_ + Cl_2(g)_ → 2/3 FeCl_3(g)_ + 4/9 O_2(g)_11.54(1)2/9 FeCr_2_O_4(s)_ + Cl_2(g)_ → 2/9 FeCl_3(g)_ + 4/9 CrCl_3(s)_ + 4/9 O_2(g)_54.03(2)1/4 MgCr_2_O_4(s)_ + Cl_2(g)_ → 1/4 MgCl_2(l)_ + 1/2 CrCl_3(s)_ + 1/2 O_2(g)_71.09(3)1/4 MgAl_2_O_4(s)_ + Cl_2(g)_ → 1/4 MgCl_2(l)_ + 1/2 AlCl_3(g)_ + 1/2 O_2(g)_78.47(4)2/3 FeO_(s)_ + Cl_2(g)_ → 2/3 FeCl_3(g)_ + 1/3 O_2(g)_−27.93(5)1/3 Fe_2_O_3(s)_ + Cl_2(g)_ → 2/3 FeCl_3(g)_ + 1/2 O_2(g)_19.53(6)1/3 Cr_2_O_3(s)_ + Cl_2(g)_ → 2/3 CrCl_3(s)_ + 1/2 O_2(g)_80.33(7)MgO_(s)_ + Cl_2(g)_ → MgCl_2(l)_ + 1/2 O_2(g)_9.57(8)1/3 Al_2_O_3(s)_ + Cl_2(g)_ → 2/3 AlCl_3(g)_ + 1/2 O_2(g)_85.93(9)

An important point of the thermodynamic findings is that the thermodynamic reactivity of the complex constituents of the chromite in chlorine is decreasing according to the following sequence:

Fe_3_O_4_ > FeCr_2_O_4_ > MgCr_2_O_4_ > MgAl_2_O_4_ (10)

**Figure 5:**
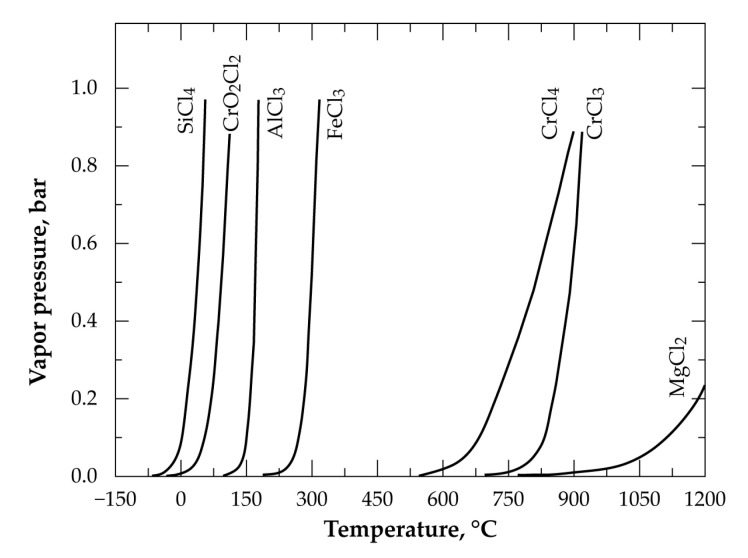
Vapor pressure versus temperature for several chlorides likely to be produced during chromite chlorination.

The evolution of the vapor pressure of chlorides of main chromite elements (Cr, Fe, Mg, Al, Si) is displayed in Figure 5 [28,29]. It indicates that in the case of the chlorination of chromite constituents, a selective separation of the obtained chlorides is feasible thanks to great differences in their vapor pressure in a selected temperature interval. A special case is the high volatility of chromyl chloride (CrO_2_Cl_2_), which will be discussed in Section 3.3.

**Figure 6:**
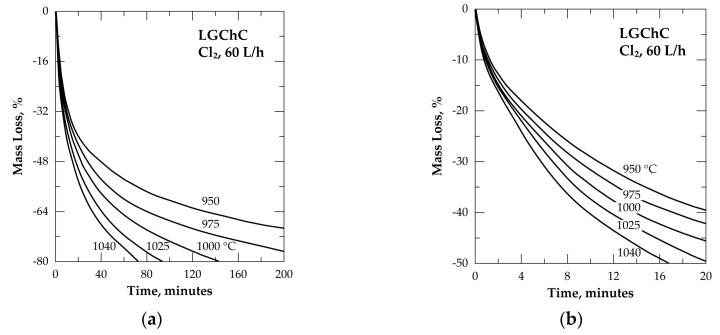
Mass change of the sample versus time for the treatment of LGChC in chlorine from 950 to 1040 °C: (**a**) General view of the obtained isotherms; (**b**) Zoom in on the graph up to 50% ML.

Based on these thermodynamic predictions and on the work previously performed [2], experimental tests of LGChC were conducted with chlorine alone with a flow rate of 60 L/h. The recorded data are depicted in Figure 6 as percent mass loss (% ML) of the sample over the reaction time. The somewhat atypical curve shape is a first indication of the complexity of the reactions of chromite with chlorine. As shown by Figure 6b, the first 50% of the sample was quickly chlorinated and volatilized, while the remaining sample appeared more refractory to chlorine. As an example, the isothermal data indicates that 32 min were sufficient to achieve 50% conversion at 950 °C while 75% conversion required 180 min at this temperature.

**Figure 7:**
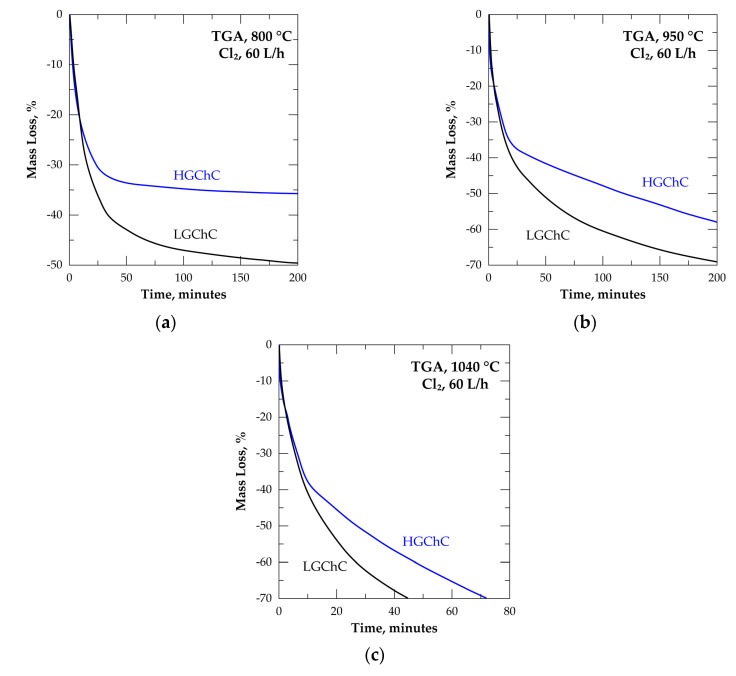
Comparison of the thermal behavior of LGChC and HGChC in Cl_2_ atmosphere: (**a**) 800 °C; (**b**) 950 °C; (**c**) 1040 °C.

To get an insight about this change in the curve shape, it is helpful to compare several isothermal TGA plots for both samples, i.e., LGChC and HGChC, as illustrated in Figure 7. The chlorination of chromite concentrates at 800 °C (Figure 7a) tends to an asymptote of the %ML beyond 2 h of treatment corresponding to about 50% and 35% ML for LGChC and HGChC, respectively.

Gathering this data with the mineralogical composition of chromite concentrates (Figure 4) and the thermodynamic predictions allows us to hypothesize that only magnetite and iron chromite are chlorinated at 800 °C. It was evaluated that these compounds (Fe_3_O_4_+FeCr_2_O_4_) represent close to 52.7% and 35.3% of the LGChC and HGChC, respectively. With this evidence, one may also conclude that the TG analysis of chromite reactions with chlorine at low temperature can be an effective method for the fair determination of the amount of (Fe_3_O_4_+FeCr_2_O_4_) contained in the chromite ores and/or concentrates. Although less wide, the difference between the %ML obtained for the LGChC and HGChC is still evident at 950 °C (Figure 7b) and 1040 °C (Figure 7c).

**Figure 8:**
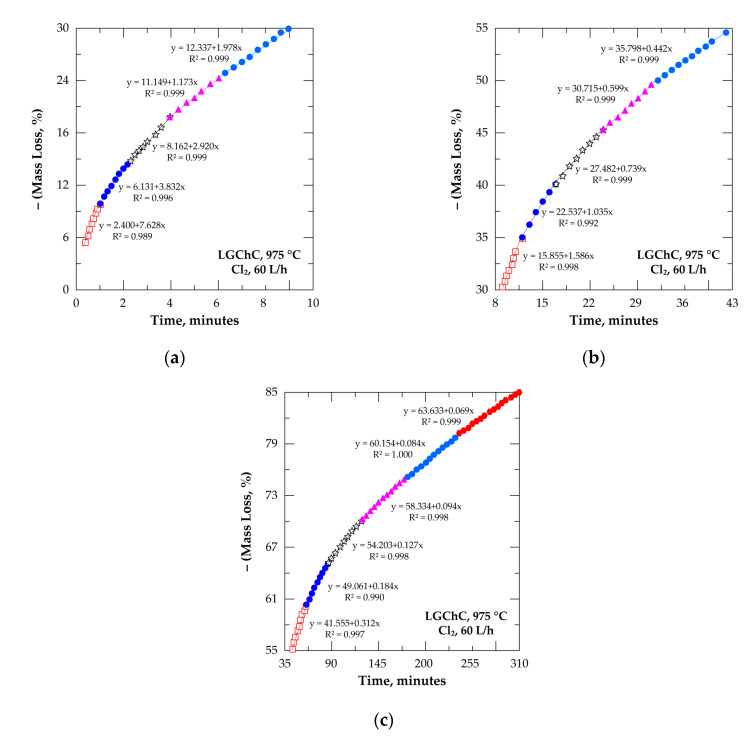
Plot of %ML versus time gathered with mean reaction rates calculated in increments of 5% ML during treatment of LGChC in chlorine at 975 °C: (**a**) 5–30% ML; (**b**) 30–55% ML; (**c**) 55–85% ML. The color markers are used to distinguish the segments of the %ML curves for which the linearization has been made for the calculation of the reaction rate.

The direct application of well-known kinetics models [30] for describing the reaction progress and rate expression faces certain difficulties related to successive reactions, altering of the physical and chemical reactivity of the remaining sample, inter-reaction between gaseous reaction products and the working sample, etc. Hence, it was suggested that the best way to evaluate the temperature impact on the chromite reactions with Cl_2_ was to calculate the reaction rate in increments of 5% mass losses in the interval ranging from 5.0% to 85.0% ML. This is performed for all isothermal data from 950 to 1040 °C. Shown in Figure 8 is the graphical representation of the processed data at 975 °C. Besides data linearization expressing the reaction rate, the correlation coefficient (R^2^) of data fitting, for each segment of 5% ML, is also shown. 

It was stated [2] that the reaction of chlorine with chromite concentrates generated metal chlorides having volatilization rate higher than their formation rate, indicating the %ML of the sample expresses directly the fraction of the sample (α) reacted. Therefore, the Arrhenius diagrams displaying the logarithm of the reaction rate plotted versus the inverse of the temperature for each 5% ML segment were drawn and values of the apparent activation energy (E_a_) with standard error were computed.

**Figure 9:**
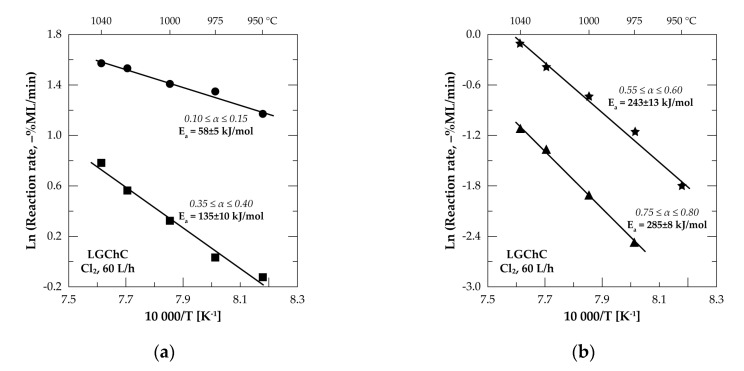
Examples of the Arrhenius diagrams for the reaction of LGChC with Cl_2_: (**a**) 0.10 ≤ α ≤ 0.15 and 0.35 ≤ α ≤ 0.40; (**b**) 0.55 ≤ α ≤ 0.60 and 0.75 ≤ α ≤ 0.80.

Figure 9 gives Arrhenius’ plots for the four chosen reacted fractions. Good fitting of the traced data is obtained with the value of E_a_ increasing from 58±5 kJ/mol at (0.10 ≤ α ≤ 0.15) to 285 ± 8 kJ/mol at (0.75 ≤ α ≤ 0.80) with a good confidence level. Furthermore, the reaction rate at 1000 °C for (0.75 ≤ α ≤ 0.80) is decreased by around 27 times with respect to the reaction rate for (0.10 ≤ α ≤ 0.15) at the same temperature, which seems unusual for the gas-solid reactions with gasification of reaction products and without formation of new solid products.

**Figure 10:**
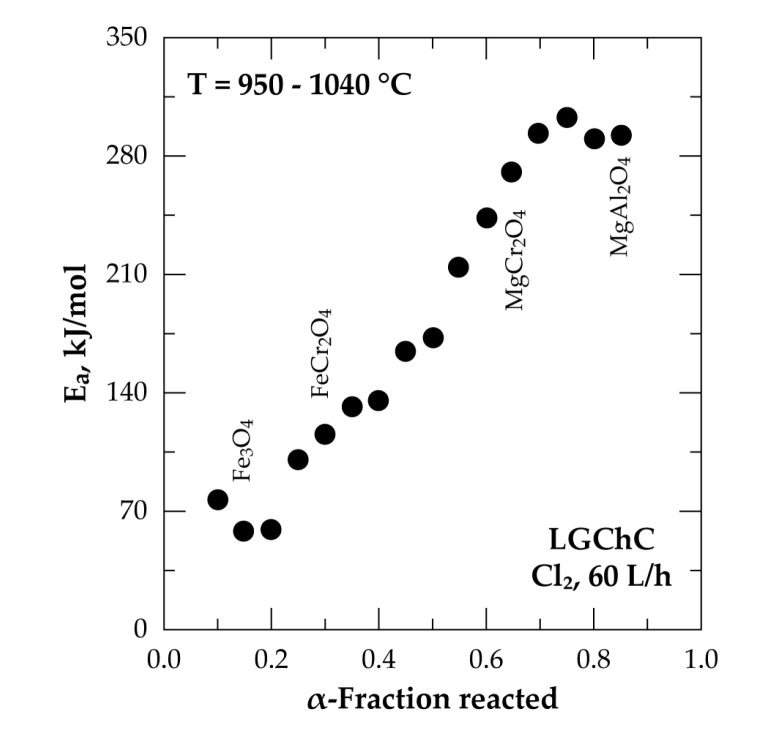
Plot of the apparent activation energy (E_a_) versus fraction reacted for the treatment of LGChC in chlorine atmosphere between 950–1040 °C.

An overall profile of the evolution of E_a_ as a function of the chromite conversion fraction is displayed in Figure 10. The beginning of the reaction proceeded with an E_a_ near to 78 kJ/mol and decreased to about 60 kJ/mol at 0.10 ≤ α ≤ 0.20. Such a trend suggests the formation of an intermediate species, unfortunately unknown, decreasing the potential barrier of the reaction. Based on the chemical and mineralogical composition of the LGChC the fraction converted at the beginning of reaction may be attributed to the reaction of Fe_3_O_4_ with Cl_2_ involving ferric chloride as final product as it is highly volatile at this temperature range (Figure 5). Thereafter, an increase of the E_a_ is observed reaching about 175 kJ/mol at (0.45 ≤ α ≤ 0.55). One may attribute globally this value of E_a_ to the reaction of iron chromite (FeCr_2_O_4_) with chlorine. Next, at α > 0.60, the apparent activation energy increased again reaching values as high as 304 kJ/mol for α ranging from 0.70 to 0.75. This conversion fraction corresponds most probably to the removal of MgCr_2_O_4_ from the chromite body. According to obtained apparent activation energy and reaction rate trends, the decreasing reaction rank of chromite constituents with chlorine follows the sequence:

Fe_3_O_4_ > FeCr_2_O_4_ > MgCr_2_O_4_ > MgAl_2_O_4_ (11)

In other words, a higher reaction rate was associated with a lower value of E_a_ and vice versa. It is hence concluded that the reactivity of chromite constituents towards chlorine is in good agreement with the apparent activation energy and the reaction rates are sufficiently different to achieve a selective elimination of one constituent without affecting the other constituents. Another point is also to be mentioned that this reaction sequence (Equation (11)) seems to match well with the sequence based on thermodynamic predictions and shown in (Equation (10)).

### 3.3. Reactions of Chromite with Chlorine in Presence of Oxygen

Having obtained information on the reaction of chromite with chlorine, it was useful to investigate the impact of oxygen on the reaction kinetic and involved products. The chemical reactions of the two main chromite constituents, FeCr_2_O_4_ and MgCr_2_O_4_, with chlorine +oxygen can be described by Equations (12) and (13), respectively. The values of Δ_r_G° are still positive, but they are much lower than those obtained for the chlorination with chlorine alone (Equations (2) and (3), respectively).

Δ_r_G° (900 °C), kJ/mol Cl_2_
1/2 FeCr_2_O_4(s)_ + Cl_2(g)_ + 3/8 O_2(g)_ → 1/4 Fe_2_O_3(s)_ + CrO_2_Cl_2(g)_5.89(12)1/2 MgCr_2_O_4(s)_ + Cl_2(g)_ + 1/4 O_2(g)_ → 1/2 MgO_(s)_ + CrO_2_Cl_2(g)_36.37(13)1/2 Cr_2_O_3(s)_ + Cl_2(g)_ + 1/4 O_2(g)_ → CrO_2_Cl_2(g)_19.47(14)

One reaction of particular interest in the system Cr-O-Cl is that of the chromium trioxide (Cr_2_O_3_) with chlorine in presence of oxygen with overall reaction described by Equation (14). As shown, the reaction consumes oxygen leading to the formation of CrO_2_Cl_2_ as final reaction product. The computed value of Δ_r_G° (900 °C) is 19.47 kJ/mol instead of 80.33 kJ/mol for the chemical reaction of Cr_2_O_3_ with chlorine solely generating CrCl_3_ at the same temperature (Equation (7)).

**Figure 11:**
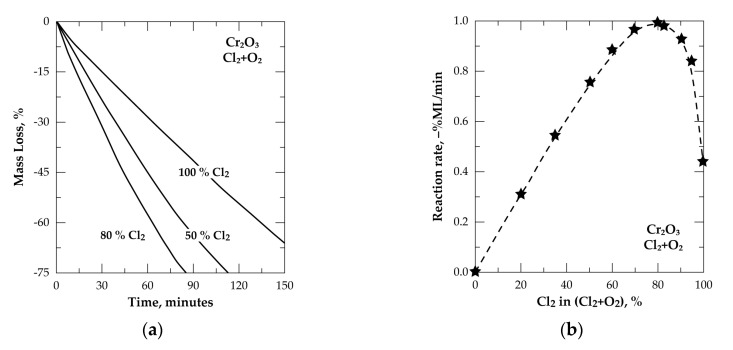
Treatment of Cr_2_O_3_ in Cl_2_+O_2_ at 800 °C: (**a**) Evolution of the sample mass loss versus time; (**b**) Dependency of the initial reaction rate on the chlorine content in the Cl_2_+O_2_ gas mixture.

This thermodynamic assessment is completed with a kinetic study of the Cr_2_O_3_ and Cl_2_+O_2_ interaction using TG isothermal tests and varying the chlorine content from 0% to 100% Cl_2_. Three typical TGA curves at 50%, 80% and 100% Cl_2_ are plotted in Figure 11a as evolution of %ML versus reaction time. More than 160 min were required to reach 75% of the Cr_2_O_3_ sample reacted in Cl_2_ alone, while the reaction time is decreased to about 112 min when an equimolar (50% Cl_2_+50% O_2_) gas mixture was used and decreased again to about 85 min when the chlorine content in the Cl_2_+O_2_ was 80%. Data displayed in Figure 11b demonstrates that the initial reaction rate (0.05 ≤ α ≤ 0.40) of the Cr_2_O_3_ chlorination with Cl_2_+O_2_ had a maximum value at 80% Cl_2_ corresponding to Cl_2_ to O_2_ ratio equal to 4. Such a result matches well with the stoichiometric coefficients of the reaction described by Equation (14) resulting in chromyl chloride as final reaction product.

According to this analysis, the LGChC is treated under a flowing gas (Cl_2_+air) with total flow rate of 61 L/h containing 28 L/h of chlorine and 33 L/h air (i.e., 26 L/h N_2_ and 7 L/h O_2_) corresponding to Cl_2_/O_2_ molar ratio equal to 4. Complementary data for choosing this gas mixture composition to enhance the chromium oxide reaction rate and to lower the reaction of iron oxides with the chlorinating gas mixtures are found in previously published research reports [31,32,33,34].

**Figure 12:**
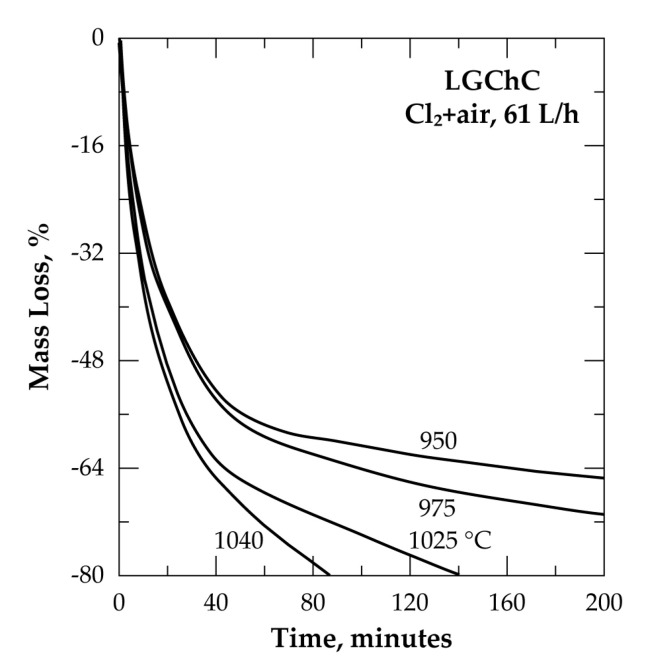
Mass change of the sample versus time for the treatment of LGChC in Cl_2_+air.

Shown in Figure 12 is the %ML versus time for the isothermal treatment of the LGChC under the above-mentioned atmosphere between 950 and 1040 °C for reaction time up to 200 min. As in the previous cases (Figure 5 and Figure 6), there is an abrupt change on the curve shape after 50% ML, it is more obvious at low temperature and reflected on the reaction progress. For instance, the first 50% ML was reached with a reaction time of 36 min at 950 °C, while more than 410 min are needed to reach 75% ML of the sample.

**Figure 13:**
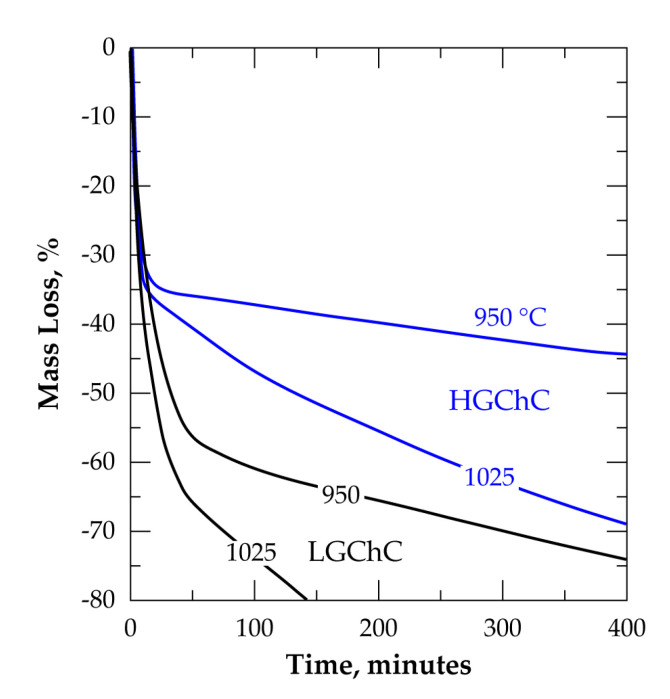
Comparison of the isothermal data for LGChC and HGChC treatment at 950 and 1025 °C under chlorine in presence of oxygen.

For comparison, the acquired TG data for the reactions of LGChC and HGChC with chlorine in presence of oxygen are shown in Figure 13. The main observations are the high difference between %ML of HGChC and LGChC and the slope change of the %ML vs. time (ML rate), which is clearly more pronounced at 950 °C for both materials, although this evidence is also visible at 1025 °C. Combining the chemical and mineralogical composition of both concentrates with these isoconversion data lead to assign the kinetic changes to the substantial reaction of MgCr_2_O_4_ with chlorine for the conversion higher than 35% and 50 % for HGChC and LGChC, respectively. Both thermodynamic and kinetics reactivity may explain this particular behaviour of magnesiochromite in the Cl_2_+air gaseous mixture.

**Figure 14:**
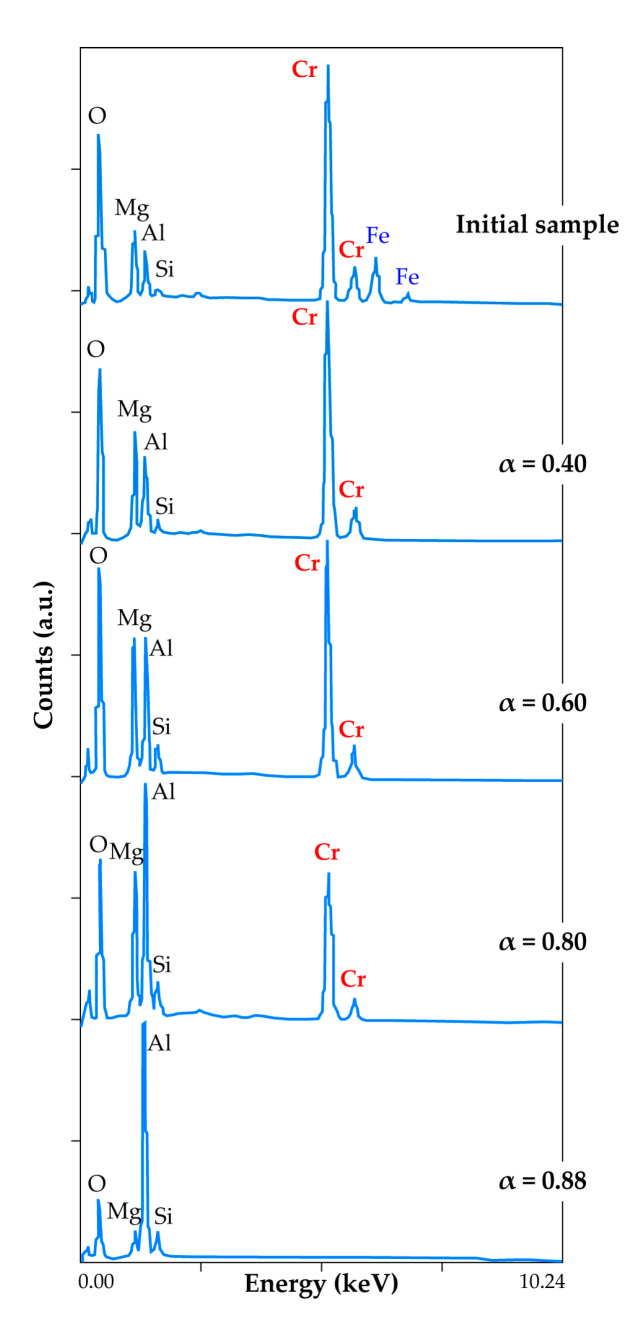
SEM-EDS results of residue from the oxychlorination of HGChC at different α-values.

To have an idea about the evolution of the elemental and mineralogical composition of the treatment residue, the HGChC reacted at various α-values was examined by SEM-EDS (Figure 14). This method of analysis was preferred to XRD due to the presence of chromite crystalline isomorph phases.

Distinct characteristic peaks of Cr, Mg, Al, Fe, Si and O are present in the SEM-EDS spectrum of the initial sample, in good agreement with the HGChC elemental composition afore-mentioned in Figure 3. The product corresponding to the fraction reacted α = 0.40 does not contain iron. As iron bearing compounds of the chromite body are FeO·Fe_2_O_3_ and FeO·Cr_2_O_3_, the SEM-EDS analysis of the fraction reacted at α-0.40 is an indirect confirmation for the removal of magnetite and iron chromite from the HGChC. Spectra at α = 0.60 and α = 0.80 with their decreasing chromium peak intensity reflect the evolution of the composition, down to a chromium-free residue at α = 0.88. Accordingly, magnesio-chromite (MgCr_2_O_4_) had reacted at 0.35 < α < 0.88, while the true spinel (α = 0.88) appeared more refractory to chlorine in presence of oxygen. Reasoning by analogy, the reaction pathways of the LGChC with chlorine in presence of oxygen will be similar, with the conversion fraction agreeing with its mineralogical composition (15.1% Fe_3_O_4_, 37.6% FeCr_2_O_4_, 26.4% MgCr_2_O_4_, 20.8% MgAl_2_O_4_) displayed in Figure 4. The reaction rates are higher due to the fast reaction of 52.7% (Fe_3_O_4_+FeCr_2_O_4_) with chlorine, hence exhibiting more reactive surface for the progress of the reaction.

**Figure 15:**
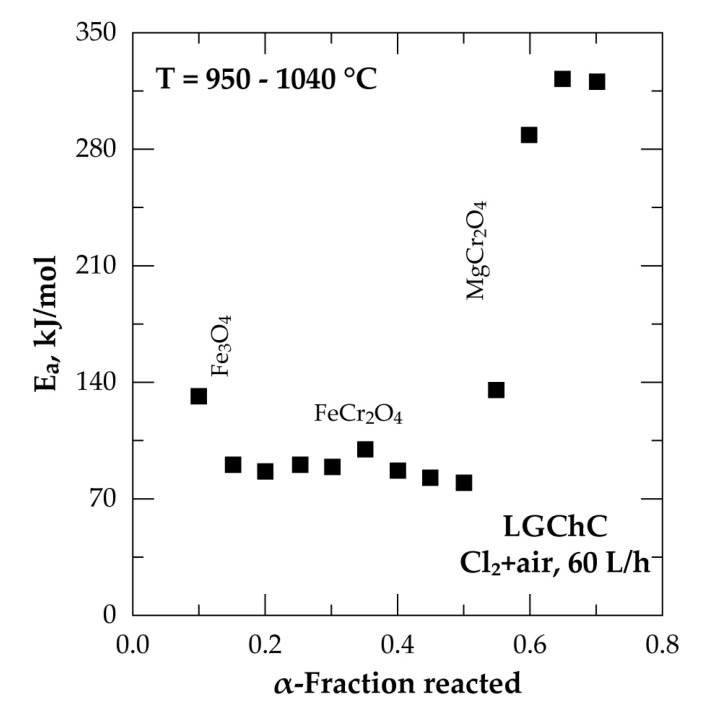
Plot of the apparent activation energy (E_a_) versus fraction reacted for the treatment of LGChC with Cl_2_+air at 950–1040 °C.

The Arrhenius plot shown in Figure 15 for the reaction of LGChC with Cl_2_+air at various α-values illustrates large changes in the apparent activation energy, starting from about 130 kJ/mol at beginning of the reaction, followed by an average E_a_ of about 80–85 kJ/mol at 0.15 ≤ α ≤ 0.50 and by a final strong increase up to 300 kJ/mol.

**Figure 16:**
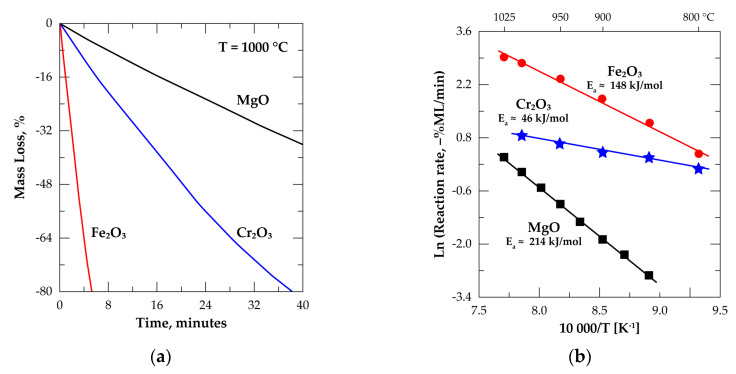
Treatment of Fe_2_O_3_, Cr_2_O_3_ and MgO in chlorine in presence of oxygen: (**a**) Evolution of the sample mass loss versus time at 1000 °C; (**b**) Arrhenius diagrams between 800 and 1025 °C.

To help to interpret these peculiar changes, isothermal treatments under Cl_2_+O_2_ (Cl_2_/O_2_ = 4) of the main oxides (Fe_2_O_3_, Cr_2_O_3_ and MgO) of the chromite constituents (Fe_3_O_4_, FeCr_2_O_4_ and MgCr_2_O_4_) were performed up to 1025 °C. Note that ferrous oxide (FeO) is transformed into FeCl_3_ and Fe_2_O_3_ under a chlorinating atmosphere [32]. The experimental data showed that the reactivity of these oxides towards Cl_2_+O_2_ is widely different. As an example, 90% of the Fe_2_O_3_ sample was reacted for 10 min at 950 °C. This reaction time, for reaching the same reaction extent, was extended to 60 min and 270 min for Cr_2_O_3_ and MgO, respectively at 950 °C. As shown in Figure 16a, this trend of the TGA measurements is still evident during the treatment of these oxides at 1000 °C. Based on the isothermal data recorded, the Arrhenius plots for the reactions of the above-mentioned oxides with Cl_2_+O_2_ are reported in Figure 16b. The E_a_ values are about 148, 46 and 214 kJ/mol for the Fe_2_O_3_, Cr_2_O_3_ and MgO reactions, respectively. In addition, the decreasing reaction rate ranking of simple oxides with Cl_2_+O_2_ follows the sequence represented by Equation (15).

Fe_2_O_3_ > Cr_2_O_3_ > MgO (15)

Assuming that the chromite constituents (FeO·Fe_2_O_3_, FeO·Cr_2_O_3_, MgO·Cr_2_O_3_) are chlorinated only when both constituents (FeO and Fe_2_O_3_; FeO and Cr_2_O_3_; MgO and Cr_2_O_3_) are chlorinated and taking into account that the whole reaction rate of each chromite constituent is governed by the slowest reaction rate of its simple constituent, one may deduce that:

- the first compounds to be reacted are iron oxides of Fe_3_O_4_, the value of E_a_ obtained for Fe_2_O_3_ reaction are close to that obtained for the beginning of the chromite reaction with Cl_2_+O_2_,
- the reaction of chromite with Cl_2_+O_2_, for 0.15 ≤ α ≤ 0.50, is controlled by the reaction rate of Cr_2_O_3_ (contained in FeCr_2_O_4_) being the slowest step of the overall reaction rate. The lower value of E_a_ seems another argument for Cr_2_O_3_ impact on the overall reaction of chromite,
- the rest of the chromite reaction with Cl_2_+O_2_ (α > 0.50) is affected by the reaction of MgO (MgCr_2_O_4_) which is characterized by a low reaction rate and a high E_a_ value.

However, the energy of the chemical binding of the simple constituents in the chromite structure may affect the values of the inherent activation energy and the multistep reaction rates.

The comparison of the kinetic parameters for the reaction of chromite with chlorine and Cl_2_+air showed a difference in the apparent activation energy values (Figure 10 and Figure 15) essentially for α between 0.20 and 0.55. Two factors may explain that. First, from a thermodynamic point of view, the reaction of Cr_2_O_3_ with Cl_2_ (Equation (7)) is less favourable than that with Cl_2_+O_2_ (Equation (14)); the E_a_ values accordingly appear higher for the FeCr_2_O_4_ chlorination with Cl_2_ alone. Second, the increasing and higher apparent E_a_ with Cl_2_ alone may reflect a MgCr_2_O_4_ reaction, characterized by a high E_a_, starting before the FeCr_2_O_4_ reaction is finished. 

In spite of these differences, the present study showed an atypical temperature impact on the chlorination of chromite due to the combination of different thermodynamic and kinetics aspects of chromite component reactions with chlorine in absence and/or presence of the oxygen.

**Figure 17:**
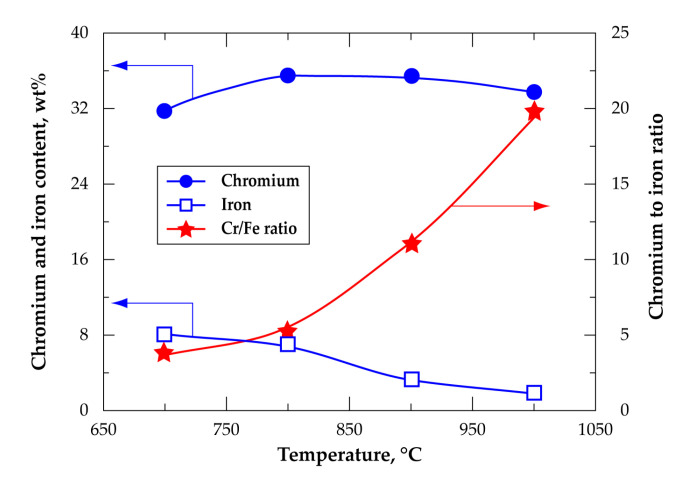
Evolution of chromium and iron content and chromium to iron ratio versus temperature during treatment of HGChC in a Cl_2_+air gaseous mixture.

As described in the previous sections, TGA tests were performed with small powder samples (40–50 mg) and high flowrates (e.g., 60 L/h Cl_2_) of reactive gases to attenuate the reaction starvation impact and to enhance mass and heat transfers. To be closer to the practical chlorination process, tests using tenth grams of chromite concentrates were also performed in a horizontal setup described earlier [2] under Cl_2_+air atmosphere from 700 to 1000 °C. The obtained data are depicted in Figure 17 as evolution of the chromium and iron content and chromium to iron ratio of the residues showing the function of the treatment temperature. These results show the preferential removal of iron from 700 °C; about 77% of iron and 18% of chromium were extracted during the treatment of HGChC at 900 °C for 2 h. The chromium and iron contents of the obtained residue at 900 °C were 35.6 and 3.2 wt%, respectively. As shown in Figure 17, the chromium to iron ratio increased rapidly from 3.9 at 700 °C to reach values as high as 11.1 at 900 °C.

To gain understanding of the physical state of the chromium bearing phase synthetized during reaction of chromite with chlorine in presence of oxygen, a two-step cooling of the outlet gases was performed, first at room temperature and second at much lower temperature (−35° C).

**Figure 18:**
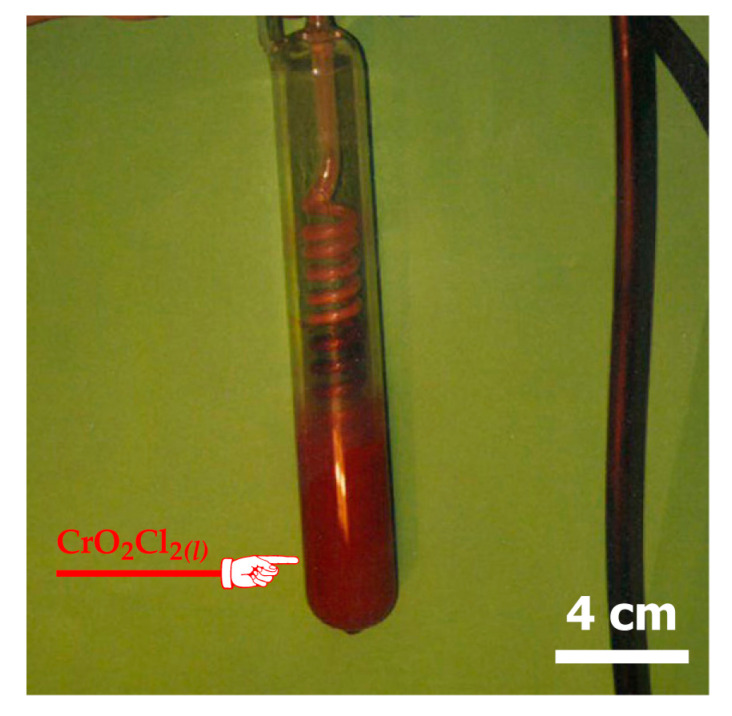
Visual image of the CrO_2_Cl_2(l)_ generated during the thermal treatment of chromite by Cl_2_+air and collected in liquid state at –35 °C.

The analyses of the solid condensate obtained at room temperature by SEM-EDS technique indicated the absence of chromium in the solid phase. However, a red liquid was isolated and collected (Figure 18) in a glassware vessel emerged in a refrigerated alcohol bath. This liquid produced reddish brown fumes in air and seems to correspond to chromyl chloride (CrO_2_Cl_2_) characteristics containing chromium at hexavalent state and it is characterized by a high vapor pressure at room temperature (Figure 5). The CrO_2_Cl_2_ synthesis was also reported in other research works [35,36,37,38,39,40] during thermal treatment of various chromium bearing materials.

By analogy, the ability of chlorine to oxidize iron (II,III) into iron(VI) in high alkali medium by gas-solid and solid-solid reactions was also demonstrated in recent investigations [41,42,43,44,45,46].

This research work gave several insights for the evolution of the (Fe,Cr,Mg,Al)-O-Cl system, in the case of chromite, at different temperatures. Nonetheless, more in-depth and detailed studies are needed to complete the current knowledge in such a complex system.

## 4. Conclusions

Thermogravimetric analysis technique provides valuable information to fairly evaluate the constituent composition of complex materials such as chromite and to analyze its reactions with Cl_2_ and Cl_2_+O_2_ gaseous mixtures.

The reactions of (Fe,Mg)(Cr,Al,Fe)_2_O_4_ constituents, for both chromite concentrates (low grade chromite concentrate-LGChC and high grade chromite concentrate-HGChC) with chlorine in isothermal conditions proceeded by gradual scheme starting by the reaction of iron oxides (Fe_3_O_4_) followed by interaction of iron chromite (FeCr_2_O_4_). Magnesio-chromite (MgCr_2_O_4_) appeared stable in Cl_2_ and Cl_2_+O_2_ atmosphere at temperatures equal to or lower than 800 °C. 

The overall reaction of LGChC with chlorine is affected differentially by temperature at 950–1040 °C, resulting in an apparent activation energy strongly dependent on the degree of conversion, e.g., increasing sharply from about 60 kJ/mol to 300 kJ/mol for fractions reacted of 0.15 and 0.75, respectively. Having a low reactivity, the MgCr_2_O_4_ compound required high temperature for the reaction to occur. Similar trends were observed for the reaction of chromite with chlorine in presence of oxygen although the values of the apparent activation energy are somewhat different.

Thermodynamic analysis of the envisaged reactions of the chromite constituents with Cl_2_ and Cl_2_+O_2_ gave complementary elements for further clarifying this particular behavior of chromite in the chlorinating atmosphere.

The kinetics results of the simple oxides (Fe_2_O_3_, Cr_2_O_3_, MgO) reactions with Cl_2_+O_2_ in the interested temperature range was another insightful building block for better understanding the multistep process of chromite processing under chlorine in presence of oxygen.

Low temperatures and short times for the interaction chromite-chlorine favor the preferential removal of iron from the low-grade concentrate, giving a chromite with a chromium-to-iron ratio satisfactory for the ferrochrome production. The presence of oxygen in the system favors the synthesis of pure chromyl chloride.
